# Financing and Delivering Pre-Exposure Prophylaxis (PrEP) to End the HIV Epidemic

**DOI:** 10.1017/jme.2022.30

**Published:** 2022

**Authors:** Amy Killelea, Jeremiah Johnson, Derek T. Dangerfield, Chris Beyrer, Matthew McGough, John McIntyre, Rebekah E. Gee, Jeromie Ballreich, Rena Conti, Tim Horn, Jim Pickett, Joshua M. Sharfstein

**Keywords:** HIV, PrEP, Drug Pricing, Access, Federal Policy

## Abstract

The U.S. has the tools to end the HIV epidemic, but progress has stagnated. A major gap in U.S. efforts to address HIV is the under-utilization of medications that can virtually eliminate acquisition of the virus, known as pre-exposure prophylaxis (PrEP). This document proposes a financing and delivery system to unlock broad access to PrEP for those most vulnerable to HIV acquisition and bring an end to the HIV epidemic.

## Executive Summary

The U.S. has the tools to end the HIV epidemic, but progress has stagnated. A major gap in U.S. efforts to address HIV is the under-utilization of medications that can virtually eliminate acquisition of the virus, known as pre-exposure prophylaxis or PrEP.

Fewer than 25 percent of individuals with PrEP indications according to Centers for Disease Control and Prevention (CDC) guidelines actually receive a PrEP prescription. Among those who do receive a prescription, adherence is also challenging. Individuals most at risk for HIV acquisition are least likely to have reliable access to PrEP. There are also enormous disparities across race, ethnicity, gender, and geography.

This document proposes a financing and delivery system to unlock broad access to PrEP for those most vulnerable to HIV acquisition. A national PrEP program would provide access to medications using a federal procurement strategy coupled with state and local implementation. It would also support access to needed laboratory services where there are no other sources of payment. The system would move away from the current reliance on high-cost, brand-name drugs that have resulted in overly complex, difficult-to-navigate programs for the uninsured and a relatively small number of access points in the Medicaid program.

A national PrEP program would dramatically and equitably expand PrEP access today and create a platform for the effective and rapid deployment of novel PrEP medications tomorrow. In doing so, it would help put the national effort to end the HIV epidemic on track to reduce new infections by 90 percent by 2030. Moreover, by developing a network of frontline community health organizations, a national PrEP program would accelerate efforts to address other public health emergencies, including COVID-19 and the opioid overdose crisis.

## Overview

In 2019, nearly 37,000 people in the U.S. were diagnosed with HIV. Black and Latinx/Hispanic individuals comprised 42 percent and 29 percent of new diagnoses, respectively.^1^ Every person living with HIV requires a lifetime of treatment at an estimated individual cost of about $501,000, with potential adverse effects that include liver toxicity, and insulin resistance.^2^ HIV was the underlying cause of death for more than 5,000 people in 2019 in the U.S.^3^

The HIV epidemic can be stopped. In 2019, the federal government launched a major new initiative called Ending the HIV Epidemic, investing more than $500 million in HIV prevention, treatment, and research programs. The goal is to reduce new HIV infections in the United States by 90 percent by the year 2030.^4^ Though achievable, success will require substantial improvement over the modest 9 percent decline in new diagnoses from 2015 to 2019.^5^

**Figure 1:**
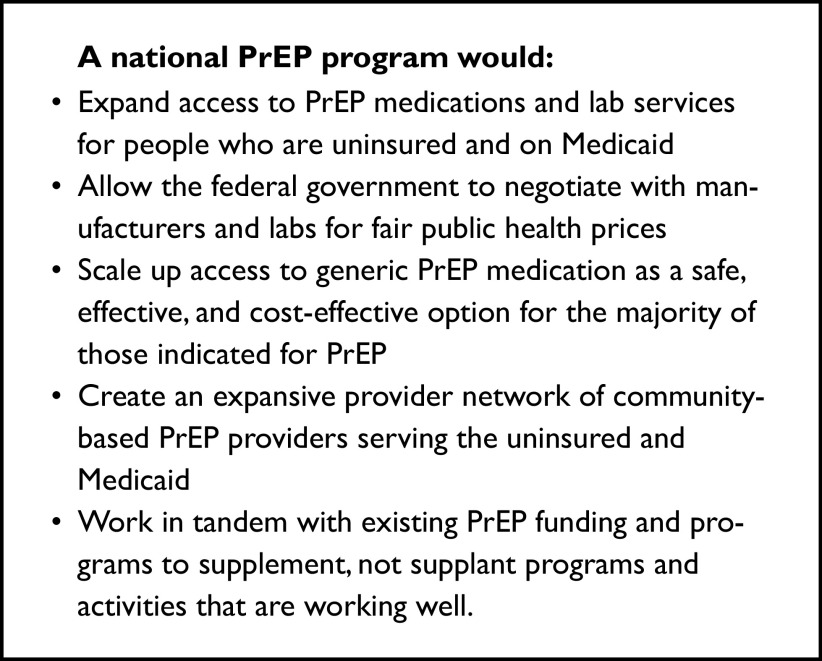
Overview of National PrEP Program

A major gap in U.S. efforts to address HIV is the under-utilization of medications that can virtually eliminate acquisition of the virus.^6^ The preventive use of these medications is known as pre-exposure prophylaxis, or PrEP.^7^ Only 25 percent of people with an indication for PrEP receive it,^8^ with large disparities by race, ethnicity, gender, and geography. In 2020, the CDC found that 66 percent of White Americans recommended for PrEP received a prescription, compared to 16 percent of Latinx/Hispanic Americans and just 9 percent of Black Americans.^9^

This document proposes a PrEP financing and delivery system to unlock broad access for those most vulnerable to HIV acquisition.

PrEP access is complex, and there are many reasons beyond financing challenges for low utilization in the U.S. These include stigma, low HIV risk perceptions, lack of provider understanding of PrEP, and other social determinants of health that impact access to a wider range of public health and health care services. A more effective national PrEP financing and delivery system alone will not solve these important issues. It can, however, serve as a mechanism for addressing these issues more effectively.

This is a policy proposal to help put the U.S. on track to end the HIV epidemic.

## Background

### 1. PrEP is effective — and cost effective

First approved by the Food and Drug Administration (FDA) in 2012, PrEP medications are antiretroviral medications that can be taken regularly to prevent acquisition of HIV.^10^

There are now two approved PrEP combinations for oral administration: emtricitabine/tenofovir disoproxil fumarate, known as TDF/FTC, and emtricitabine/tenofovir alafenamide, known as TAF/FTC. Their indications are similar, with the exception that TAF/FTC is not FDA-approved for cisgender women.^11^ These medications are about 99 percent effective at preventing acquisition of HIV from sex and at least 74 percent effective at preventing acquisition of HIV from injection drug use.^12^ In addition to their high effectiveness, these medications are generally safe when used as directed.^13^

A third medication, long-acting injectable cabotegravir marketed by ViiV Healthcare under the brand name Apretude, is an injectable formulation taken every two months approved by the FDA in December 2021.^14^ The FDA approved Apretude based on successful clinical trials showing increased adherence. This administration route will likely have advantages for some individuals.^15^ There are other products, including monthly pills, in the development pipeline.^16^

The CDC has estimated that successful expansion of PrEP access, in combination with other interventions, can be expected to prevent as many as 1 in 5 new HIV infections each year.^17^ Other countries that have dramatically scaled up access to PrEP have demonstrated even more significant reductions in HIV incidence.^18^ Because of this potential, along with expanded access to testing and treatment, PrEP access is a core pillar of the national Ending the HIV Epidemic initiative.^19^

People taking PrEP also require ongoing access to a set of recommended laboratory services, including tests for HIV, kidney function, and sexually transmitted infections.^20^ Stable access to care is essential to effective PrEP use, because stopping the medications prematurely is associated with increases in HIV risk.^21^

PrEP can also be highly cost effective. TDF/FTC is available in low-cost, generic form, with prices as low as $26/month for a 30-day supply — compared to an approximately $1,900 list price per month for branded TAF/FTC.^22^ Because lower cost generic PrEP is safe and effective for the vast majority of individuals, expansion of PrEP access can be affordable and cost-saving to the healthcare system.^23^ Similarly, ensuring that new PrEP products are available at fair prices to all those who need them will ensure cost-effective and sustainable access as new medications are approved.

### 2. PrEP is under-utilized, with large disparities by race and ethnicity

Despite the strong evidence of PrEP effectiveness in preventing HIV infections, only 25 percent of people who are indicated for PrEP have actually received a prescription.^24^ The rate of use is far below the 50 percent target set as a federal benchmark for the Ending the HIV Epidemic initiative.^25^ The number of new PrEP users further dropped during the COVID-19 pandemic.^26^

**Figure 2:**
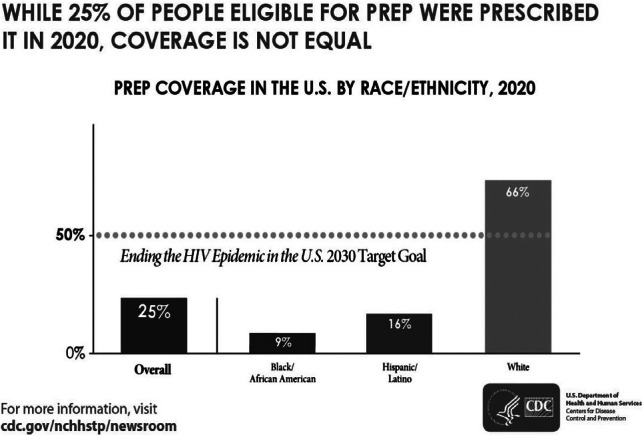
PrEP Coverage by Race/Ethnicity, 2020

There are alarming and growing disparities by race and ethnicity in who is aware of and prescribed PrEP. (Figure 2). White individuals are over seven times more likely to use PrEP than Black individuals and over four times more likely to use PrEP than Latinx/Hispanic individuals.^27^ Despite greater HIV incidence among Black and Latinx gay, bisexual, and other sexual minority men (SMM),^28^ White SMM are significantly more likely to report PrEP awareness, discussion with a health care provider, and use.^29^ These disparities also track geographic lines, with the South accounting for only 30 percent of PrEP users, but more than half of new HIV diagnoses.^30^

Not only is access to PrEP fairly low overall, there also continue to be challenges in achieving optimal PrEP persistence (i.e., consistent use of PrEP over time) with wide variations depending on type of payer and race/ethnicity.^31^

Finally, there are also major gaps based on gender, with PrEP use nearly three times as high among men than among women, and based on gender identity, with only three percent of sexually active transgender people using PrEP.^32^

### 3. PrEP access in the U.S. for people without health insurance is fragmented, complex, and inadequate

Since 2012, when the first PrEP product was approved by the FDA, the PrEP landscape has been dominated by two brand-name products manufactured by Gilead Sciences — Truvada (TDF/FTC) and Descovy (TAF/FTC), with list prices of about $1,800 and $1,900 per month respectively in January 2022.^33^

These high initial prices put a robust, centralized public health response to PrEP access for the uninsured out of reach. In the U.S., there is broad access to treatment for people with HIV through the federal Ryan White HIV/AIDS Program. However, there is no similar comprehensive public health safety net for PrEP access. When PrEP was first approved — with impressive safety and efficacy — the nation should have been engaged in a widespread national campaign to build demand for this new product. But the prohibitively high price made only a limited PrEP rollout possible.

Instead, access to PrEP has come through a patchwork of overlapping and often confusing manufacturer assistance programs, state PrEP assistance programs, and federal efforts. (Table 1).

**Table 1:** Fragmented Access to PrEP for Uninsured People in the U.S.

Program/Pathway	Medication	Labs	Provider network limitations
**Gilead Advancing Access (manufacturer assistance program)** ^**1**^	Truvada and Descovy	No lab services or other PrEP ancillary services are covered	Broad pharmacy distribution network
**Ready, Set, PrEP (federal PrEP program)**^**2**^	Truvada and Descovy	No lab services or other PrEP ancillary services are covered	More limited pharmacy distribution network
**State PrEP Assistance Programs (limited to handful of states)**^**3**^	Generally, no drug benefit; refer to Gilead patient assistance program or Ready, Set, PrEP for medication (Truvada or Descovy)	Most state PrEP assistance programs cover lab services and clinic visits	Limited clinical provider network
**340B PrEP programs**	Purchase drugs at 340B discount; financial incentive for 340B providers to prescribe Truvada and Descovy over generic TDF/FTC	Most 340B PrEP providers cover lab services and clinic visits, and outreach and linkage services, but 340B revenue used to provide these services is diminishing	Access depends on availability of local 340B provider with a PrEP program
**CDC HIV prevention funding**^**4**^	CDC policy prohibits use of most HIV prevention funding for PrEP “medications, clinical care, or labs other than HIV or viral hepatitis screening”	As of November 2021, CDC policy now allows HIV prevention funding to be used for PrEP ancillary services, including labs.	Wide network of HIV prevention providers (community-based organizations, STD clinics, and health departments) funded to provide PrEP outreach, education, and other ancillary services

Table References

1. Gilead Advancing Access, “Get Started with Gilead Advancing Access Program,” *available at* <https://www.gileadadvancingaccess.com/get-started-advancing-access> (last visited March 16, 2022*).*

2. Department of Health and Human Services, “Ready, Set, PrEP: Find Out If You Qualify to Enroll for Free PrEP Medications,” *available at* <https://readysetprep.hiv.gov> (last visited March 16, 2022*).*

3. NASTAD, “State PrEP Assistance Programs,” *available at* <https://www.nastad.org/prep-access/state-prep-assistance-programs> (last visited March 16, 2022).

4. National Association of County and City Health Officials, “PrEP Ancillary Support Services Now Allowable Use of CDC HIV Funding,” January 3, 2022, *available at* <https://www.naccho.org/blog/articles/cdc-release-guidance-that-up-to-15-of-a-state-city-awards-can-be-used-for-prep-ancillary-service> (last visited March 16, 2022*).*

Programs for the uninsured still remain centered on brand-name manufacturer assistance and donation programs and predominantly favor brand-name products. For example, the federal safety net program for PrEP launched in 2019, known as Ready, Set, PrEP, depends on a substantial donation of Truvada and Descovy by Gilead Sciences.^34^ It notably does not include any other product, despite the fact there are currently 11 manufacturers marketing generic TDF/FTC in the U.S. and ViiV Healthcare marketing long-acting cabotegravir under the brand-name Apretude.^35^

The complexity disproportionately impacts communities that historically lack easy access to health care systems due to intersecting systems of racism and oppression. The same fault lines of socioeconomic status, access to housing, and access to insurance that impact a range of health outcomes also affect access to PrEP. And these challenges are even more dire for populations with the least access to health care services, including undocumented individuals, those who are incarcerated and leaving incarceration, people who inject drugs, and people experiencing homelessness.

PrEP assistance programs come with frequent eligibility checks, which can be difficult and frustrating for providers and individuals alike. Initial enrollment also requires multiple steps and forms of identification, which may present additional barriers to access for patients.^36^ The complexity of obtaining PrEP for the uninsured also means there are relatively few access points in many states.

Many clinics that might be able to provide prescriptions struggle with offering patients help in signing up for the myriad drug and other assistance programs. Those that provide PrEP for the uninsured must employ dedicated staff just to handle the burden of the paperwork for these programs. It is also true that in many places, larger 340B clinical entities are the only PrEP game in town because they are the only entities able to navigate the complexity of PrEP financing. Through the Gilead Advancing Access program, these providers have received reimbursement in excess of acquisition cost for uninsured patients. Even then, under the best of circumstances, some individuals stop using PrEP to avoid the hassle of repeated demonstrations of need.^37^

The complex approach to access for the uninsured is contrary to the evidence demonstrating that easy, rapid access is needed for this population. A recent study found that nearly 1 in 5 people prescribed PrEP did not pick it up at initiation at the pharmacy.^38^ Studies consistently find that interventions that deliver medications quickly (referred to as “low threshold”) — such as drop-in visits, same-day PrEP,^39^ streamlined testing, standing orders for labs, and 90-day prescriptions — correlate with greater PrEP uptake and persistence.^40^ These models include pharmacy-based PrEP and mobile PrEP programs.^41^ The latter are particularly important for unstably housed individuals and people who inject drugs.

The limitations of this fragmented system are also apparent in gaps in access to required laboratory services. For people without health insurance, laboratory access largely depends on the capacity of state and local programs to cover these services. Individual health departments are left to negotiate these PrEP lab prices with commercial labs, leading to wide variability in prices. As a result, many people cannot access PrEP because of lack of access to necessary laboratory tests.^42^ During consultation with men who now or previously used PrEP for the development of this proposal, one man said of laboratory costs, “It’s kind of expensive for a disease I don’t have.”^43^

The toll that complexity takes on access is severe and could compound other barriers to PrEP, including stigma. Health care providers must take on the uncompensated administrative task of helping consumers navigate multiple programs, diverting resources from other tasks and programs. Individuals are also faced with learning about several different coverage programs, as well as an array of applications and enrollment pathways that require a time commitment that many simply do not have. An uninsured consumer said, “I stopped using [PrEP] because it became too much of a hassle to keep verifying my information every month. That I didn’t have a job, that I didn’t have income. And it started making me feel bad,”^44^ underscoring the consequences of complex application and eligibility processes.

The complexity disproportionately impacts communities that historically lack easy access to health care systems due to intersecting systems of racism and oppression.^45^ The same fault lines of socioeconomic status, access to housing, and access to insurance that impact a range of health outcomes also affect access to PrEP. And these challenges are even more dire for populations with the least access to health care services, including undocumented individuals, those who are incarcerated and leaving incarceration, people who inject drugs, and people experiencing homelessness.^46^

### 4. PrEP access can be improved in the Medicaid program

The Medicaid expansion under the Affordable Care Act has provided access to essential health care for millions of Americans. The expansion, which now extends to 38 states, has been associated with modest improvements in PrEP access.^47^ Medicaid programs are entitled to statutorily mandated discounts through the Medicaid Drug Rebate Program and have also been able to secure supplemental discounts offered by manufacturers, which have helped to facilitate access to PrEP medications.

However, despite this progress, many people enrolled in Medicaid still do not access PrEP services. One reason for slow uptake is hesitancy to prescribe among primary care clinicians.^48^ As another consumer said, “Doctors don’t really know much about PrEP. Like how to prescribe it or how much it would cost me or how I can get it covered.” Another consumer explained, “I go to an LGBT health clinic for my PrEP because primary care doctors really don’t know anything about PrEP.” ^49^ Stigma may also play a part, even among doctors who care for HIV patients.^50^

Variability in clinical engagement can translate into inconsistent PrEP programs. As one consumer put it, “They need to establish a consistent protocol for when you can actually get PrEP. Because some doctors won’t give it to you until you get your labs back, some doctors only give you a 30-day supply, and others will let you have 90.”^51^ Moreover, effective clinical innovations, such as same-day PrEP ^52^ are also relatively rare.^53^

In some states, an additional barrier facing patients covered by Medicaid are gaps in access to laboratory testing. Even in states with high HIV incidence, recommended testing for people receiving PrEP, such as regular testing for gonorrhea and chlamydia, may not be covered. ^54^ In other states, self-testing options for sexually transmitted infections are not covered, or even permitted.^55^ While it is too early to tell how Medicaid programs will approach Apretude coverage, given the high price tag and the availability of other options for PrEP, we could see similar confusing and variable prior authorization and other utilization management techniques as have been applied to Descovy.

The end result is that many people covered by Medicaid lack reliable access to PrEP. A CDC study showed that in 2018, Medicaid provided far fewer PrEP prescriptions than private insurers.^56^ And for those able to get PrEP, the access appears to be less consistent. A CDC study found that uninterrupted PrEP use was 13.7 months among those commercially insured compared to only 6.8 months among those on Medicaid.^57^ In the Medicaid group, Black individuals had the shortest PrEP persistence compared with White and other race/ethnicity groups at 4.7 months compared to 7.3 and 8.0 months, respectively.^58^ In theory, this difference could be due to differences in appropriate PrEP use. A more likely explanation is less consistent access to PrEP.

### 5. There are few PrEP access points in community settings, limiting access for people who are uninsured or on Medicaid

A fundamental challenge in expanding access to PrEP is that many people who need PrEP — whether uninsured or on Medicaid — may not have regular sources of medical care at all. These individuals would benefit from PrEP access in a broad range of community settings such as HIV prevention outreach programs, mobile units, domestic violence shelters,^59^ drug treatment facilities, pharmacies, and health departments. According to a 2020 national survey, fewer than half of local health departments are engaged in promoting PrEP, and fewer than 1 in 5 are able to provide PrEP starter packs.^60^

Evidence is emerging that telehealth programs offer tremendous advantages for PrEP access and may be able to bridge some of these gaps in community settings.^61^ Telehealth programs for PrEP are increasing across the country, driven in part by adaptations of HIV services in response to the COVID-19 pandemic.^62^ Some programs, such as Iowa’s, have established a “telePrEP” program through a partnership between the state’s health department and an academic medical center.^63^ Other states are partnering with new PrEP mail order companies to ensure access to medication and PrEP labs through mail order and home testing.^64^

Combined with expansion of pharmacy-based PrEP programs,^65^ telehealth programs are able to expand the availability of PrEP in areas where there may not be a traditional clinical provider and are well positioned to partner with community outreach programs and extend into rural communities. However, these programs are not at a scale to reach a significant number of people.

The consequences of these gaps are evident in the limited initial success of the federal Ready, Set, PrEP program. Despite Ready, Set, PrEP’s ambitious goal of covering 10,000 uninsured individuals in its first year, as of June 2020, the program had provided prescriptions for only about 800 people.^66^ These gaps also help explain the enormous racial and ethnic disparities in PrEP access. Reducing disparities will require meeting more people with PrEP access where they are.

### 6. Underuse of generic PrEP medications limits PrEP access and increases costs

The U.S. failure to provide broad access to PrEP in part reflects the lack of a strategy for using low-cost, generic medications. The cost and complexity of medication access makes it difficult to make PrEP available in new settings. Meanwhile, where people do access PrEP, there is evidence of overuse of the expensive brand-name medication Descovy.^67^ Apretude may present the same ethical and financial dilemmas when it comes to access. Apretude’s list price sits at 72 times that of generic TDF/FTC and researchers are already questioning whether the medication is cost effective at that price.^68^

Consumers want access to safe and effective medications and may rely on prescribers to help guide those decisions. As one consumer said, “Generic is fine as long as the side effects aren’t worse. Y’all just need to make sure it’s accessible at different places like mobile units and pharmacies.”^69^

This dynamic creates an opportunity for policy. A coherent U.S. strategy for PrEP can be at once more accessible and more affordable. Financing and delivering care in a way that makes the most use of low-cost, generic medications offers the potential to expand access to care dramatically. Such a system would also position the U.S. well to make the most effective use of new versions of PrEP for those who need them.

## A Policy Proposal

Expanding access to PrEP in the U.S. requires a new approach. To develop this proposal, the authors consulted with more than 30 experts in HIV, pharmaceutical, and laboratory policy, federal partners, and governmental public health leaders along with PrEP consumers.^70^

This project was supported by a grant from Arnold Ventures to the Johns Hopkins Bloomberg School of Public Health.

### 1. The federal government should establish a federal program for PrEP medications and laboratory services to reach people who are uninsured or covered by Medicaid

We propose a national PrEP program to enhance access for people who are uninsured or covered by Medicaid.^71^ The program should involve direct federal purchase of PrEP medications and lab services and engagement of a broader network of qualified and community facing providers, with state and local participation to support community access to PrEP.

The program should aim to achieve six goals:

- **Accessibility.** PrEP should be available through a large network of access points able to meet people where they are. In addition to community health centers and other essential health care providers, access points should include a wide range of community-based programs, including mobile outreach, drug treatment programs, programs that address intimate partner violence and transgender health access, local health departments, corrections programs, and pharmacies. Same day PrEP starts should be as widely available as possible.
- **Equity.** PrEP should be widely available in communities most vulnerable to HIV acquisition and most affected by access barriers, including Black and Latinx/Hispanic communities.
- **Simplicity.** PrEP should be easy to access from the point of view of the individual and easy to administer from the point of view of the clinical or community-based program. A national program should not disrupt existing successful efforts.
- **Affordability.** PrEP and associated laboratory services should be available at no cost to individuals.
- **Sustainability.** An extended federal commitment to PrEP financing and delivery depends on the broad adoption of low-cost, generic medications. Federal bulk purchase of PrEP medications and laboratory services can create the greatest value for invested funds.
- **Adaptability.** A national PrEP program should provide a foundation and infrastructure that is able to adapt to new formulations of PrEP and to other efforts to counter public health challenges.

A national PrEP program should support greater access to care both in the health care system and in nontraditional community settings. It should also serve as a platform to accelerate HIV prevention efforts, including a national PrEP awareness campaign and technical assistance and education for health providers. Moreover, the network of outreach programs brought together to support PrEP access could be utilized to reach populations at high risk of other serious health concerns.

In this sense, the proposal aims for more than putting medications in the hands of people who need it. It aims to build a more resilient system of care delivery to increase access, equity, and health.

The national PrEP program would consist of three parts: the bulk purchase and distribution of medications through pharmacies (Part A), options to expand PrEP access in clinical settings (Part B), and a new network of nontraditional community sites supported by telemedicine (Part C). (Table 2).

**Table 2:** Overview of a National PrEP Program

Part A	A national bulk purchase of PrEP medications with availability through a large pharmacy network for people who are uninsured or covered by Medicaid. Access at the pharmacy should be seamless for the consumer.
**Part B**	Options for clinical settings to (1) provide on-site dispensing and (2) offer laboratory services for those without coverage. These opportunities can allow clinics to provide PrEP more frequently and effectively.
**Part C**	A national network of nontraditional community sites to offer PrEP, supported by telehealth. This network can reach people who do not regularly access clinical health services.

#### Part A: Purchasing and Making Available PrEP Medications

The federal government should establish a streamlined federal purchasing mechanism for PrEP medication to obtain a stable supply at a low price. For a national PrEP program, the CDC could secure a large bulk purchase or subscription model with manufacturers.

Given the value of low-cost, generic PrEP, the initial focus would be on these products. There are currently 11 manufacturers marketing generic TDF/FTC in the U.S. at a price around $26 for a 30-day bottle of medication in January 2022.^72^ A federal bid for manufacturer contracts would have the ability to leverage bulk purchasing power to negotiate a competitive price for mass purchase of generic TDF/FTC products. Such a bid should be structured to engage multiple generic companies, reducing the possibility of supply disruptions.

To make these medications broadly available, the federal government should contract with a broad network of pharmacies, using an arrangement consistent with usual pharmacy operations. For example, the program can work on a replenishment or “virtual stock” model, in which the pharmacy distributes PrEP from existing “neutral” inventory. The pharmacy then identifies which individuals are covered by Medicaid or uninsured. The pharmacy then can bill the national PrEP program at the negotiated price for the drug as well as for the dispensing fee.

The global purchase will assure individuals on Medicaid access to PrEP without a co-pay, while adding broad access for those who are uninsured.

*Alternative medication*. Some individuals are unable to take low-cost, generic PrEP medications for medical reasons, such as renal insufficiency.^73^ For these individuals, the alternative medication of TAF/FTC is preferable. Based on evidence-based guidelines, the federal program can make this medication available through the same network of pharmacies, combining a bulk purchase with the federal government’s ten-year contract with Gilead Sciences for the donation of TAF/FTC. Similarly, with the approval of Apretude, individuals who struggle with adherence to once-daily pills could benefit from a long-acting injectable option. A bulk order or global purchase combined with a set of evidence-based guidelines can ensure that access is based on clinical need.

If manufacturers do not participate in a national contract, and other options are not available, providers could still refer people in need of alternative medications to traditional avenues of access.

#### Part B: Options for the Clinical System to Provide On-Site Dispensing and Laboratory Services for Those without Coverage

To enhance access to PrEP in clinical settings with on-site prescribers, the national PrEP program should offer two options: on-site dispensing and coverage for laboratory services for those without coverage.

*On-site dispensing.* Clinicians should be able to order PrEP medications from a distributor to support same-day starts, an approach to care that increases the probability of PrEP use. These providers can, in essence, have a bottomless “PrEP cabinet” on site, under a set of policies for access and security set by the national program.

To make this option possible, the federal program should contract with a distributor who can purchase PrEP medications at the federally negotiated price.

*Laboratory services for the uninsured and underinsured.* Clinicians should be able to access laboratory services for patients who do not now have a source of payment. These patients can be sent to a “laboratory network of last resort,” with data returned in formats easily integrated with electronic health records.

To make this option possible, the federal program should contract with a national laboratory or laboratories to provide covered services using a fee schedule. The contract should require laboratories to make results available electronically to clinicians. At least one option in the lab network should be for self-testing in states where self-testing is permitted.

Through these two options, clinicians — including Medicaid providers — can choose to enhance PrEP access for their patients.

#### Part C: A Broad Network of Nontraditional Community Sites for PrEP Access Supported by Telehealth

To broaden PrEP access substantially, a national PrEP program should engage community service providers that reach people at the highest risk for HIV acquisition. This strategy is especially important to close racial, ethnic, and rural disparities in access to PrEP.

The first component of this effort is a broad network of community partners willing to serve as PrEP access points. This network should start with CDC HIV prevention grantees, such as outreach programs, mobile prevention units, domestic violence shelters, drug treatment centers, and others. These programs should receive additional grant support to (1) educate and train their staff on PrEP and (2) establish a mechanism to connect clients with telehealth providers for PrEP access. States should develop this network to assure it is responsive to the needs of diverse communities at risk for HIV.

The second component is the authorization of a limited number of telehealth providers for PrEP in each state. These programs should be able to screen people for PrEP need, prescribe PrEP (using the pharmacy network of the program and relying on evidence-based clinical criteria), manage laboratory services (using the laboratory network of the program), and provide follow-up. Standards of care could include referrals to other social service programs and to health care clinics for primary care and other services, including referral to STD treatment if needed.

Opportunities to provide telehealth should be made available to traditional medical providers. 340B clinics (including community health centers), pharmacies, and other local providers should be eligible to apply to serve as the authorized telehealth providers in their states. The federal program and states should work together to authorize these providers. The federal program should manage this limited network by setting standards and permitting billing for clinical services through a national fee schedule.

The third component is the linking of the broad community partner network to the telehealth providers. Each community partner should be linked to one telehealth provider, with an opportunity to switch at designated points in time to improve service. In this way, for example, a program that works with survivors of intimate partner violence can consistently link participants to a PrEP telehealth program. Long-acting injectable formulations of PrEP may require a different set of staffing and administration considerations than oral formulations and innovative delivery models should be assessed and integrated into the national PrEP program.

### 2. A national PrEP program should meet the goals of consumers, clinics, community organizations, pharmacies, states and localities, and the federal government

**Consumers** should see easy access to PrEP through pharmacies as well as greater access to laboratory services. Many consumers should have new access to same-day prep from clinics that take the option for on-site dispensing. Consumer access to PrEP should also expand dramatically at nontraditional community locations.

**Healthcare providers in a clinical setting** should gain options to expand access to PrEP, with little interference in existing operations. They can opt in for same-day dispensing on site and to refer patients to a laboratory network of last resort. They can expand PrEP services with confidence that they will be able to care for all their patients. Some larger clinical programs, such as community health centers, may choose to become telehealth providers to support access to care in nontraditional locations.

**Community-based organizations** should have a new service to offer: linkage to PrEP on the spot through telehealth. This opportunity should provide value to the organizations and their clients. Expanded CDC HIV prevention funding should be used to scale up capacity of these providers.

**Telehealth providers** will have major new opportunities to partner with community sites to make PrEP available, with reimbursement for clinical services off of a fee schedule.

**Pharmacies** should dispense PrEP to people who are uninsured and covered by Medicaid using existing mechanisms and receiving an appropriate dispensing fee. Pharmacies should also have opportunities to anchor or participate in new telehealth operations.

**States** should have the opportunity to build a network of community partners to offer PrEP in nontraditional settings, relying on local knowledge and community engagement. They should also play a central role in selecting telehealth providers to support care provision in these settings.

The **federal government** should benefit from the substantial increase in PrEP access and reduction in HIV infection. The program should also open a window into trends in PrEP access, creating new visibility into progress ending the HIV epidemic.

### 3. A national PrEP program should broaden access to innovative PrEP medications as they are approved for use

The first long-acting injectable form of PrEP, cabotegravir, was approved by the FDA in December 2021 and there are additional PrEP products in the research and development pipeline. This long-acting formulation may be preferable for certain individuals who are unable to take medication daily. However, with the list price set at $22,500 per year, there is a high risk that this medication and others that follow will suffer the same fate as the original PrEP medications: high prices and limited access with major missed opportunities for HIV prevention. A national PrEP program should aim to broaden access to long-acting, injectable, cabotegravir. This could be accomplished using a bulk purchase linked to a set of evidence-based clinical criteria for use. It could also be accomplished through a national subscription model, which would permit unlimited access for qualified individuals for a set payment to the manufacturer. The draw for manufacturers to participate in this program would be a functioning, large-scale PrEP delivery system, with a network of community providers able to offer patients rapid access to care.

### 4. A national PrEP program should avoid burdensome eligibility determinations

Burdensome and repeated eligibility determinations are undermining access to PrEP today and should not be recapitulated in a new national model for PrEP access. Instead, eligibility processes should be designed to meet the needs of individuals who are uninsured and enrolled in Medicaid.

There are a small number of privately insured Americans who also should benefit from this program. Under the Affordable Care Act, PrEP is a preventive service available without cost sharing for most individuals with private insurance. For privacy reasons, however, a small number of privately insured Americans may not be able to access PrEP through their usual coverage.

Seeking to maximize access to childhood vaccines, the Vaccines for Children program developed an approach to eligibility determinations that is a potential model for a national PrEP program. This approach sets standards for providers, rather than complex eligibility determinations for individuals.^74^ Providers are instructed to screen patients for eligibility and not to provide free vaccine to children who are known to have private coverage.

Similar to the provider network for Vaccines for Children, the provider network for a national PrEP program should focus on the populations in need. Providers would screen patients for eligibility based on federal eligibility standards.

A national PrEP program should not adopt the strict “payer of last resort” requirements that are a hallmark of the Ryan White HIV/AIDS Program. The Ryan White program relies on purchase and delivery of brand-name and expensive anti-retroviral medications as well as a clinical care model based on specialty care in infectious disease.^75^ It also involves a population of those diagnosed with a life-long chronic condition who are more likely to navigate a burdensome determination process.

Rather, a more relevant model for eligibility determinations is community-based HIV prevention programs. Programs engaged in outreach activities do not ask people to provide extensive documentation before providing essential services, including information about immigration status.^76^ By hewing to the HIV prevention paradigm, a national PrEP program would balance accessible public health service delivery with encouragement and support for public health providers to leverage public and private payers where available.

### 5. A national PrEP program should partner with state and local health departments and meaningfully engage communities most impacted by HIV

A national PrEP program should be overseen by a federal agency, while partnering with programs housed within state, territorial, and local health departments who are recipients of CDC’s HIV Surveillance and Prevention flagship funding program where possible.^77^

Key roles for state and local partners include communicating on PrEP access with the public, publicizing the options under Part B with health care providers, and identifying innovative access points for Part C. Partners may be able to help select telemedicine providers to match with community access points. If there is no state or local governmental public health capacity to perform these roles, they can be handled by a selected nongovernmental organization or by the federal agency itself.

The success of a national PrEP program depends on meaningful engagement of communities most impacted by HIV. Federal partners and state and local health departments should be expected to address community feedback and input as the national PrEP program is developed and implemented to ensure the program is responsive to community needs and concerns. At the same time, federal standards and oversight should ensure that no state is left behind in PrEP access expansion.

### 6. The core elements of a national PrEP program should permit a more efficient use of federal resources

Because of the difficulty accessing data on drug rebates, it is difficult to ascertain the full federal expenditures for PrEP medication and laboratory services across the country. It is likely that the federal government currently spends at least several hundred million dollars for PrEP access for uninsured and Medicaid populations. Reallocating existing federal resources into a national PrEP program should add significant value for the federal government.

Much of the value would accrue to the Medicaid program. The federal government is currently paying for 90 percent of costs for the Medicaid expansion population, the Medicaid eligibility category that captures most people indicated for PrEP based on CDC guidelines. Reducing unnecessary use of more expensive medications could generate significant savings to support the overall effort.^78^

Major savings would also accrue to Medicare, as fewer people would go on to develop HIV. The estimated discounted lifetime cost for people who acquire HIV at age 35 is $501,000 in FY 2019 dollars.^79^

The costs of a national PrEP program could be divided into fixed costs and variable costs based on the number of people receiving access to care. Fixed costs, which would include administrative expenses, can be estimated at approximately $100 million a year. These funds would cover CDC and state health departments and a national distributor for medications for same-day distribution.^80^

Variable costs would depend on the quantity of medications, laboratory services, and telehealth consultations provided. The expected cost of medication and dispensing for low-cost generic PrEP medications is $50/month. The cost of PrEP laboratories can be estimated at $600/year, which is also $50/month. Finally, the cost of telehealth consultations can also be estimated to be approximately $600/year (or $50/month as well).

Not all patients would require all three services from the national PrEP program:

- A first group would only utilize the program for medication costs. This group would include everyone with Medicaid coverage and accessing care through physician offices, community health centers, and other programs with Medicaid-authorized prescribers. Cost per month: $50. Estimate of size of group: 60 percent of total.
- A second group would utilize the program for medication costs and laboratory services. This group would include uninsured individuals obtaining care through physician offices, community health centers, and other clinical settings. Cost per month: $100. Estimate of size of group: 20 percent of total.
- A third group would utilize the program for medications, laboratory services, and telehealth consultation. This group would include those accessing community sites linked to telehealth providers. Cost per month: $150. Estimate of size of group: 20 percent of total.

Under these assumptions, every thousand monthly prescriptions would cost the program $80,000. Six thousand monthly prescriptions would cost less than $500,000, the value of just one prevented HIV infection.

If it is assumed that half of the estimated 1.1 million people in need of PrEP will be privately insured, then the entire remaining group could obtain low-cost PrEP for the entire year for a total cost of about $500 million dollars. Meeting half of this need would cost $250 million. (Considering that many people opt for “on demand” PrEP use rather than continuous use for a year, the total cost could be substantially less.) This investment would prevent thousands of HIV infections,^81^ easily covering both the cost of the program and associated additional funding for HIV prevention initiatives.

As other PrEP medications such as injectable cabotegravir are incorporated into practice, the expenditures would increase — and so, in theory, would the benefit from greater use of more convenient therapies. The national program could help to maximize this benefit by negotiating a global purchase or subscription model that provides greater access at similar cost to the federal government than the current fragmented approach.

Realizing the full potential of a national program for PrEP access will require more than expanded access to medications and laboratory services. Also important is funding for outreach, counseling, education, and linkage services, as well as capacity building assistance for a broad network of PrEP providers.

## Other Considerations

A national PrEP Program should build upon existing programs and inspire complementary efforts to boost PrEP use and fight the HIV epidemic.

### 1. A national PrEP program should be timed with new investments in HIV prevention programs

Realizing the full potential of a national program for PrEP access will require more than expanded access to medications and laboratory services. Also important is funding for outreach, counseling, education, and linkage services, as well as capacity building assistance for a broad network of PrEP providers. One consumer group participant noted that “a lot of people also need help with obtaining affordable housing and a job. People who have HIV get a lot of social services, but there’s nothing for people who are HIV-negative.”^82^ CDC HIV prevention funding — including Ending the HIV Epidemic Initiative funding — currently covers many of these services. New resources will allow the delivery system to scale up in tandem with access to medications and laboratory services. Policy changes that would allow more flexibility for CDC HIV prevention grantees to use HIV prevention funding for PrEP would also complement a national PrEP program.

### 2. A national PrEP program should be paired with new support for PrEP 340B providers

Historically, 340B programs have financed services for people who lack insurance by not only receiving discounted medications for uninsured individuals, but by also being able to make a margin on the use of expensive, brand-name medications for insured individuals.^83^ For PrEP medications, clinics can receive these medications at a discount compared to their reimbursement by commercial insurers. This margin is funding a large swath of PrEP activities throughout the country. A strategic switch to low-cost, generic medications, however, complicates this financial model.

In what is unique to PrEP as compared to other medications, for 340B PrEP providers, Gilead’s Advancing Access program for *uninsured* individuals also offered an opportunity for providers to generate revenue. 340B providers have been able to purchase the drug at the 340B discounted price and then seek reimbursement from Gilead’s Advancing Access program at a much higher usual and customary rate.^84^ This spread has allowed many PrEP providers to provide a range of PrEP services beyond the medication, for which there are no other funding streams.

This approach to medication access for the uninsured has created two challenges. First, it has concentrated access to PrEP among providers who can access this additional revenue. Other community providers cannot generate revenue, undermining their ability to offer PrEP. Second, Gilead’s program is in transition. The company has ended this practice of reimbursing at a price higher than acquisition cost as of January 2022, which has removed the ability of 340B providers to generate revenue for uninsured patients using this program.^85^ This change could be particularly devastating for programs in non-Medicaid expansion states (including many states in the South), where uninsured populations are larger.^86^

The flux in the 340B financing system for PrEP is threatening the ability of a subset of safety net providers to provide critical HIV services. Continuing to finance PrEP access primarily through reliance on the 340B spread available through prescribing high-cost, brand-name drugs is not sustainable and will continue the fragmentation of financing that makes scaling up PrEP so difficult.

A better policy is for enhanced CDC HIV prevention funding to relieve current financing gaps across 340B PrEP providers and work in tandem with a national PrEP program to cover medications and labs for uninsured individuals.

### 3. A national PrEP program would enable simple and effective consumer education campaigns

A national PrEP awareness and education campaign is critical to ensure individuals are aware of the value of PrEP and how to access care. The complexity of the current PrEP system undermines consumer engagement and education efforts. The fragmented system of PrEP access, particularly for the uninsured, is difficult to explain, let alone navigate. The simplicity of a national PrEP program will provide a new platform from which to launch a national PrEP education campaign. Such a campaign can drive interest and uptake in HIV prevention more generally. The campaign can leverage the new network of PrEP providers, all with close ties to communities impacted by HIV, to message the availability and importance of PrEP to individuals who are not currently engaged with the health care system.

As one consumer said, “We need something that would help people know that this is available. Many people are afraid to even ask for the services they need because they are afraid that it will cost them, so it will be important for them to be made aware that it won’t.”^87^

### 4. A new PrEP program should support efforts by community health centers to make PrEP available

Through the federal Ending the HIV Epidemic initiative, community health centers have been awarded a total of $152M across FY 2020 and FY 2021 to increase capacity to provide PrEP.^88^ This investment has yielded positive results already, expanding access to PrEP for individuals served by the nation’s massive health center system.^89^ A national PrEP program should not supplant these efforts or funding. Rather, it would allow for same-day starts and greater access to laboratory services for the uninsured.^90^

### 5. A national PrEP program should support state and federal regulatory reform to expand access to innovative models of care

The national PrEP program’s forward-leaning use of telehealth to expand access to care through nontraditional community sites should create momentum for regulatory reform.

Telehealth and prescribing are largely regulated by states, and some state rules may not permit the implementation of innovative clinical models. A national PrEP program should facilitate regulatory reform by establishing best practices for PrEP access and providing guidance for rule changes to support their implementation.

A national PrEP program should also engage with the FDA to review the label for PrEP to consider including “on-demand” use, which is now recommended by several major state and local public health agencies.

### 6. A national PrEP program should accelerate efforts to establish reliable models of self-testing for laboratory services

Recommended laboratory services for people taking PrEP medications include blood tests and swabs for sexually transmitted infections. Through the use of blood spots and self-administered swabs, some pilot programs are offering PrEP labs in a single, mail-in kit.^91^ A national PrEP program should facilitate consideration of regulatory issues for these kits, so they can be both reliable and broadly adopted.

### 7. A national PrEP program should provide a foundation to address other public health emergencies

A national PrEP program can serve as a model for more efficient use of other medical products essential to public health. One potential example is naloxone, the opioid overdose-reversal agent that is often in short supply because of a confusing and complex system of purchase and distribution.

Beyond pharmaceutical policy, the network of community access points established by a national PrEP program can be mobilized for other health crises. For example, these programs can be enlisted to counter misinformation on COVID, make testing available, and help distribute vaccines. They can also be mobilized to reduce overdose, by providing education, distributing harm reduction supplies, and linking people to addiction treatment.

By building a bridge to often neglected communities, a national PrEP program could become a platform to address other major challenges to public health.

## Note

The authors acknowledge and appreciate receiving financial support for this project from Arnold Ventures.

## References

[1] Centers for Disease Control and Prevention (CDC), “HIV in the United States and Dependent Areas,” August 9, 2021, *available at* <https://www.cdc.gov/hiv/statistics/overview/ataglance.html> (last visited March 9, 2022).

[2] P.G. Farnham , et al., “Updates of Lifetime Costs of Care and Quality-of-Life Estimates for HIV-Infected Persons in the United States: Late Versus Early Diagnosis and Entry into Care,” Journal of Acquired Immune Deficiency Syndrome 64, no. 2 (2013) 183–189. Updated to year 2019 $501,000. 10.1097/QAI.0b013e3182973966 23615000

[3] Kaiser Family Foundation, “The HIV/AIDS Epidemic in the United States: The Basics,” June 7, 2021, *available at* <https://www.kff.org/hivaids/fact-sheet/the-hivaids-epidemic-in-the-united-states-the-basics> (last visited March 9, 2022).

[4] Centers for Disease Control and Prevention, “Ending the HIV Epidemic in the U.S. (EHE): Overview,” September 7, 2021, *available at* <https://www.cdc.gov/endhiv/overview.html> (last visited March 9, 2022); HIV.gov, “Ending the HIV Epidemic Funding,” July 30, 2021, *available at* <https://www.hiv.gov/federal-response/ending-the-hiv-epidemic/funding> (last visited March 9, 2022).

[5] See CDC, *supra* note 1.

[6] Centers for Disease Control and Prevention, “PrEP Effectiveness,” May 13, 2021, *available at* <https://www.cdc.gov/hiv/basics/prep/prep-effectiveness.html> (last visited March 9, 2022); Centers for Disease Control and Prevention, “Effectiveness of Prevention Strategies to Reduce the Risk of Acquiring or Transmitting HIV,” November 12, 2019, *available at* <https://www.cdc.gov/hiv/risk/estimates/preventionstrategies.html#anchor_1562942347> (last visited March 9, 2022).

[7] This proposal does not cover post-exposure prophylaxis or PEP. However, the framework could be applied to expanding access to PEP in the future.

[8] Centers for Disease Control and Prevention, “PrEP for HIV Prevention in the US,” (2022), *available at* <https://www.cdc.gov/nchhstp/newsroom/fact-sheets/hiv/prep-for-hiv-prevention-in-the-us-factsheet.html> (last visited May 18, 2022); America’s HIV Epidemic Analysis Dashboard, “PrEP coverage,” December 2020, *available at* <https://ahead.hiv.gov/data/prep-coverage> (last visited March 9, 2022).

[9] *Id.* (CDC).

[10] U.S. Food & Drug Administration, “Drug Approval Package,” August 2, 2012, *available at* <https://www.accessdata.fda.gov/drugsatfda_docs/nda/2012/021752_truvada_toc.cfm> (last visited March 9, 2022).

[11] U.S. Food & Drug Administration, “FDA Approves Second Drug to Prevent HIV Infection as Part of Ongoing Efforts to End the HIV Epidemic,” October 3, 2019, *available at* <https://www.fda.gov/news-events/press-announcements/fda-approves-second-drug-prevent-hiv-infection-part-ongoing-efforts-end-hiv-epidemic> (last visited March 9, 2022).

[12] See CDC, “PrEP Effectiveness,” *supra* note 6; and see CDC, “Effectiveness of Prevention Strategies to Reduce the Risk of Acquiring or Transmitting HIV,” *supra* note 6.

[13] HIV.gov, “Pre-Exposure Prophylaxis,” August 2, 2021, *available at* <https://www.hiv.gov/hiv-basics/hiv-prevention/using-hiv-medication-to-reduce-risk/pre-exposure-prophylaxis> (last visited March 9, 2022); Gilead*, Gilead Presents 96-week DISCOVER Trial Data Demonstrating Favorable Renal and Bone Safety Profile of Descovy for HIV PrEP in At-Risk Populations*, Press Release, March 10, 2020, *available at* <https://www.gilead.com/news-and-press/press-room/press-releases/2020/3/gilead-presents-96-week-discover-trial-data-demonstrating-favorable-renal-and-bone-safety-profile-of-descovy-for-hiv-prep-in-at-risk-populations> (last visited March 9, 2022).

[14] U.S. Food and Drug Administration, *FDA Approves First Injectable Treatment for HIV Pre-Exposure Prevention*, Press Release, December 20, 2021, *available at* <https://www.fda.gov/news-events/press-announcements/fda-approves-first-injectable-treatment-hiv-pre-exposure-prevention> (last visited March 9, 2022).

[15] HIV Prevention Trials Network, “Study Summary: HPTN 083: An HIV Prevention Clinical Trial,” August 11, 2021, *available at* <https://www.hptn.org/research/studies/hptn083> (last visited March 9, 2022); HIV Prevention Trials Network, “Study Summary: HPTN 084: Long-Acting Injectable for the Epidemic,” November 9, 2020, *available at* <https://www.hptn.org/research/studies/hptn084> (last visited March 9, 2022).

[16] AIDS Vaccine Advocacy Coalition, “The Years Ahead in Biomedical HIV Prevention Research,” September 2021, *available at* <https://www.avac.org/infographic/years-ahead-hiv-prevention-research> (last visited May 18, 2022).

[17] Centers for Disease Control and Prevention, *As Many as 185,000 New HIV Infections in the U.S. Could Be Prevented by Expanding Testing, Treatment, PrEP*, Press Release, February 24, 2016, *available at* <https://www.cdc.gov/nchhstp/newsroom/2016/croi-press-release-prevention.html> (last visited March 9, 2022).

[18] A.E. Grulich , et al., “Long-Term Protection from HIV Infection with Oral HIV Pre-exposure Prophylaxis in Gay and Bisexual Men: Findings from the Expanded and Extended EPIC-NSW Prospective Implementation Study,” Lancet HIV 8, no. 8 (2021): e486–e494; D. Colby, et al., “HIV pre-Exposure Prophylaxis and Health and Community Systems in the Global South: Thailand Case Study,” *Journal of International AIDS Society* 18 (2015): 19953. 34217426 10.1016/S2352-3018(21)00074-6

[19] Centers for Disease Control and Prevention, “Ending the HIV Epidemic in the U.S. (EHE): Prevent,” July 27, 2021, *available at* <https://www.cdc.gov/endhiv/prevent.html> (last visited March 9, 2022).

[20] Centers for Disease Control and Prevention, *Preexposure Prophylaxis for the Prevention of HIV Infection in the United States — 2017 Update: A Clinical Practice Guideline*, March 2018, at 47-48, *available at* <https://www.cdc.gov/hiv/pdf/risk/prep/cdc-hiv-prep-guidelines-2017.pdf> (last visited March 9, 2022).

[21] D.P. Serota , et al., “Beyond the Biomedical: Preexposure Prophylaxis Failures in a Cohort of Young Black Men Who Have Sex with Men in Atlanta, Georgia,” Clinical Infectious Diseases 67, no. 6 (2018): 965–970; S.E. Rutstein, et al., “Initiation, Discontinuation, and Restarting HIV Pre-exposure Prophylaxis: Ongoing Implementation Strategies,” *Lancet HIV* 7, no. 10 (2020): e721-e730. 32861269 10.1016/S2352-3018(20)30203-4 PMC7541752

[22] 46Brooklyn, “NADAC Drug Pricing Dashboard,” *available at* <https://www.46brooklyn.com/nadac> (last visited March 16, 2022).

[23] S. Gavaskar, “Expensive New Drug Could Undermine HIV Prevention Efforts,” March 9, 2020, *available at* <https://medicine.yale.edu/news-article/23020> (last visited March 16, 2022); R.P. Walensky , et al., “Comparative Pricing of Branded Tenofovir Alafenamide/Emtricitabine Relative to Generic Tenofovir Disoproxil Fumarate/Emtricitabine for HIV Pre-exposure Prophylaxis: A Cost-Effectiveness Analysis,” Annals of Internal Medicine 172, no. 9 (2020): 583–590; L.Y. Wang, et al., “Cost-Effectiveness of Pre-exposure Prophylaxis among Adolescent Sexual Minority Males,” *Journal of Adolescent Health* 66, no. 1 (2020): 100-106; D.C. Daskalakis and O. Blackstock, “TDF-FTC Is Still the First-Line Regimen for PrEP,” NYC Health, January 2020, *available at* <https://www1.nyc.gov/assets/doh/downloads/pdf/ah/first-line-regimen-prep.pdf> (last visited March 16, 2022).32150602

[24] CDC, “PrEP for HIV Prevention in the US,” *supra* note 8.

[25] Centers for Disease Control and Prevention, “HIV Testing, Treatment, Prevention Not Reaching Enough Americans,” December 3, 2019, *available at* <https://www.cdc.gov/nchhstp/newsroom/2019/ending-HIV-transmission-press-release.html> (last visited March 16, 2022).

[26] L. Tao, et al., “Real-World Utilization of F/TDF and F/TAF for HIV Pre-exposure Prophylaxis during the COVID-19 Pandemic in the US, December 2019-June 2020,” Abstract OAC0203, 11th IAS Conference on HIV Science July 18-21, 2021, *available at* <https://www.natap.org/2021/IAS/IAS_14.htm> (last visited March 16, 2022).

[27] CDC, “PrEP for HIV Prevention in the US,” *supra* note 8.

[28] Centers for Disease Control and Prevention, “HIV: African Americans,” September 23, 2021, *available at* <https://www.cdc.gov/hiv/group/racialethnic/africanamericans/index.html> (last visited March 16, 2022); Centers for Disease Control and Prevention, “HIV: Hispanic/Latino People,” October 12, 2021, *available at* <https://www.cdc.gov/hiv/group/racialethnic/hispaniclatinos/index.html> (last visited March 16, 2022).

[29] C. Yang , et al., “Awareness of and Interest in Pre-Exposure Prophylaxis among Patients Receiving Services at Public Sexually Transmitted Disease Clinics in an Urban Setting,” Journal of Health Care for the Poor and Underserved 32, no. 1 (2021): 537–549; Y.A. Huang, et al., “HIV Preexposure Prophylaxis, by Race and Ethnicity — United States, 2014-2016,” *Morbidity and Mortality Weekly Report* 67, no. 41 (2018): 1146-1150; A.J. Siegler, et al., “Policy- and County-Level Associations with HIV Pre-exposure Prophylaxis Use, the United States, 2018,” *Annals of Epidemiology* 45 (2020): 24-31. 33678712 10.1353/hpu.2021.0039 PMC8627588

[30] Centers for Disease Control and Prevention, *HIV in the Southern United States*, September 2019, *available at* <https://www.cdc.gov/hiv/pdf/policies/cdc-hiv-in-the-south-issue-brief.pdf> (last visited March 16, 2022).

[31] Y.A. Huang , et al., “Persistence with Human Immunodeficiency Virus Pre-exposure Prophylaxis in the United States, 2012–2017,” Clinical Infectious Diseases 72, no. 3 (2021): 379–385. 33527117 10.1093/cid/ciaa037

[32] J.M. Sevelius , et al., “HIV Testing and PrEP Use in a National Probability Sample of Sexually Active Transgender People in the United States,” Journal of Acquired Immune Deficiency Syndrome 84 (2020): 437–442; CDC, *supra* note 8. 10.1097/QAI.0000000000002403 PMC7340231 32692101

[33] M.L. Shaw , “In Debate Over PrEP, Researchers Raise Questions about Benefit vs Value,” AJMC, January 13, 2020, *available at* <https://www.ajmc.com/view/in-debate-over-prep-researchers-raise-questions-about-benefit-vs-value-> (last visited March 16, 2022).

[34] T. Straube , “Why Are Only 891 People Enrolled in a Free PrEP Program for 200,000?” POZ, June 2, 2020, *available at* <https://www.poz.com/article/891-people-enrolled-free-prep-program-200000> (last visited March 16, 2022).

[35] U.S. Food and Drug Administration, “Drugs@FDA: FDA-Approved Drugs,” *available at* <https://www.accessdata.fda.gov/scripts/cder/daf/index.cfm?event=overview.process&ApplNo=090513> (last visited March 16, 2022); U.S. Food and Drug Administration, “FDA Approves First Injectable Treatment for HIV Pre-Exposure Prophylaxis,” December 20, 2022, available at <https://www.fda.gov/news-events/press-announcements/fda-approves-first-injectable-treatment-hiv-pre-exposure-prevention> (last visited March 16, 2022).

[36] Gilead Advancing Access, *supra* note 34; US Department of Health and Human Services, *supra* note 35; ViiV Connect, “Patient Enrollment,” *available at* <https://www.viivconnect.com/for-providers/patient-enrollment/> (last visited March 16, 2022).

[37] K. Ming et al., “Improving the HIV PrEP Continuum of Care Using an Intervention for Healthcare Providers: A Stepped-Wedge Study Protocol,” BMJ Open 10 (2020): e040734; L.A. Eaton, et al., “Stigma and Conspiracy Beliefs Related to Pre-exposure Prophylaxis (PrEP) and Interest in Using PrEP among Black and White Men and Transgender Women Who Have Sex with Men,” *Journal of AIDS and Behavior* 21 (2017): 1236-1246.

[38] L. Dean , et al., “Novel Population-Level Proxy Measures for Suboptimal HIV Preexposure Prophylaxis Initiation and Persistence in the USA,” AIDS (London, England) 35, no. 14 (2021): 2375–2381. 10.1097/QAD.0000000000003030 PMC8564020 34723852

[39] K.F. Kamis , et al., “Same-Day HIV Pre-Exposure Prophylaxis (PrEP) Initiation During Drop-in Sexually Transmitted Diseases Clinic Appointments Is a Highly Acceptable, Feasible, and Safe Model that Engages Individuals at Risk for HIV into PrEP Care,” Open Forum Infectious Diseases 6, no. 7 (2019): 310–317. 10.1093/ofid/ofz310 PMC6641790 31341933

[40] See, e.g., N.D. Laborde , et al., “Understanding PrEP Persistence: Provider and Patient Perspectives,” AIDS and Behavior 24, no. 9 (2020): 2509–2519. 32048078 10.1007/s10461-020-02807-3 PMC8054778

[41] Y. Jo , et al., “Interest in Linkage to PrEP among People Who Inject Drugs Accessing Syringe Services; Miami, Florida,” *PLoS ONE* 15, no. 4 (2020): e0231424. A.M. Roth, et al., “Integrating HIV Preexposure Prophylaxis With Community-Based Syringe Services for Women Who Inject Drugs: Results from the Project SHE Demonstration Study,” *Journal of Acquired Immune Deficiency Syndrome* 86 (2021): e61-e70; R. Bellman, et al., “An Observational Survey Assesses the Extent of PrEP and PEP Furnishing in San Francisco Bay Area Pharmacies,” *Journal of the American Pharmacists Associations* (2021); C.M. Khosropour, et al., “A Pharmacist-Led, Same-Day, HIV Pre-Exposure Prophylaxis Initiation Program to Increase PrEP Uptake and Decrease Time to PrEP Initiation,” *AIDS Patient Care and STDs* 34, no. 1 (2020).

[42] Ming, *supra* note 41.

[43] PrEP Consumer Group, August 2021.

[44] *Id*.

[45] Eaton, *supra* note 41. G. Aidoo-Frimpong , et al., “A Review of Cultural Influences on Risk for HIV and Culturally-Responsive Risk Mitigation Strategies Among African Immigrants in the US,” Journal of Immigrant and Minority Health (2020): online; R.A. Brooks, et al., “Predictors of Awareness, Accessibility and Acceptability of Pre-exposure Prophylaxis (PrEP) among English- and Spanish-Speaking Latino Men Who have Sex with Men in Los Angeles, California,” *Journal of Immigrant and Minority Health* 22 (2020): 708-716. 10.1007/s10903-019-00955-w 31823164

[46] S.A. Golub , “PrEP Stigma: Implicit and Explicit Drivers of Disparity,” Current HIV/AIDS Reports 15 (2018): 190–197. D. English, et al., “Intersectional Social Control: The Roles of Incarceration and Police Discrimination in Psychological and HIV-Related Outcomes for Black Sexual Minority Men,” *Social Science and Medicine* 258 (2020): 113-121. 32590189 10.1016/j.socscimed.2020.113121 PMC7506501

[47] B.F. Farkhad , et al., “Effect of Medicaid Expansions on HIV Diagnoses and Pre-Exposure Prophylaxis Use,” American Journal of Preventive Medicine 60, no. 3 (2021): 335–342; D. Karletsos and C. Stoecker, “Impact of Medicaid Expansion on PrEP Utilization in the US: 2012-2018,” *AIDS and Behavior* 25, no. 4 (2021): 1103-1111. 33104923 10.1007/s10461-020-03070-2

[48] J.T. Jones , et al., “Pre-exposure Prophylaxis (PrEP) Awareness and Prescribing Behaviors among Primary Care Providers: DocStyles Survey, 2016-2020, United States,” AIDS and Behavior 25, no. 4 (2021): 1267–1275. 33201428 10.1007/s10461-020-03089-5

[49] PrEP Consumer Group, *supra* note 43.

[50] A.D. Castel , et al., “Understanding HIV Care Provider Attitudes regarding Intentions to Prescribe PrEP,” Journal of Acquired Immune Deficiency Syndrome 70, no. 5 (2015): 520–528. 10.1097/QAI.0000000000000780 PMC4644475 26247895

[51] PrEP Consumer Group, *supra* note 43.

[52] Kamis, *supra* note 43.

[53] See, e.g., Laborde, *supra* note 44.

[54] Ming, *supra* note 41.

[55] N. Seiler , Leveraging Financing and Coverage Benefits: Medicaid Strategies to Deliver PrEP Intervention Services, AcademyHealth, January 2019, *available at* <https://academyhealth.org/sites/default/files/leveragingfinancingcoveragemedicaidstrategiesprep_jan2019_0.pdf> (last visited March 16, 2022).

[56] N.W. Furukawa , et al., “National Trends in Drug Payments for HIV Preexposure Prophylaxis in the United States, 2014 to 2018: A Retrospective Cohort Study,” Annals of Internal Medicine 173, no. 10 (2020): 799–805. 32894696 10.7326/M20-0786 PMC7674258

[57] Huang, *supra* note 31.

[58] *Id*.

[59] T.C. Willie , et al., “You Never Know What Could Happen: Women’s Perspectives of Pre-Exposure Prophylaxis in the Context of Recent Intimate Partner Violence,” Women’s Health Issues 30, no. 1 (2020): 41–48; T.L. O’Malley, et al., “Intimate Partner Violence, HIV Pre-Exposure Prophylaxis (PrEP) Acceptability, and Attitudes about Use: Perspectives of Women Seeking Care at a Family Planning Clinic,” *Journal of AIDS and Behavior* 25 (2021): 427-437; Ruth Ellis Center, “What We Do,” *available at* <https://www.ruthelliscenter.org> (last visited March 16, 2022).31537431

[60] D. Smith , L. Grant , and J. Zigman , NACCHO, “Assessing the Impact of COVID-19 Response on Local Health Department HIV PrEP Implementation Activities,” National Sexual Health Conference, October 8, 2021, virtual presentation (presentation on file with author).

[61] E.E. Chasco , et al., “Bringing Iowa TelePrEP to Scale: A Qualitative Evaluation,” American Journal of Preventive Medicine 61, no. 5 (2021): S108–117. 34686280 10.1016/j.amepre.2021.05.040

[62] NASTAD, “State-Specific Tele-PreP Practices,” *available at* <https://www.nastad.org/maps/state-specific-tele-prep-services> (last visited March 16, 2022); A.J. Siegler , et al., “Developing and Assessing the Feasibility of a Home-based Preexposure Prophylaxis Monitoring and Support Program,” Clinical Infectious Diseases 68, no. 3 (2018): 501–504. 10.1093/cid/ciy529 PMC6336909 29982304

[63] PrEP Iowa, “TelePrEP,” *available at* <https://www.prepiowa.org/teleprep> (last visited March 16, 2022).

[64] See, e.g., MISTR, a company that provides telehealth access to PrEP prescribers, mail order delivery of medications, and access to home testing for PrEP labs, *available at* <https://heymistr.com/#howitworks> (last visited March 16, 2022).

[65] M.I. Lopez , et al., “Implementation of Pre-exposure Prophylaxis at a Community Pharmacy through a Collaborative Practice Agreement with San Francisco Department of Public Health,” Science and Practice Advances in Pharmacy Practice 60, no. 1 (2019): 138–144. 10.1016/j.japh.2019.06.021 31405804

[66] Straube, *supra* note 38; C. Sloan, et al., “PACHA Highlights Need to Address HIV PrEP Coverage Disparities,” Avalere, April 7, 2021, *available at* <https://avalere.com/insights/pacha-highlights-need-to-address-hiv-prep-coverage-disparities> (last visited March 16, 2022).

[67] J.L. Marcus , et al., “Switching from Tenofovir Disoproxil Fumarate to Tenofovir Alafenamide for Human Immunodeficiency Virus Preexposure Prophylaxis at a Boston Community Health Center,” Open Forum Infectious Diseases 8, no. 8 (2021): ofab372. 10.1093/ofid/ofab372 PMC8496521 34631926

[68] N. Brown , “Study Supports Pricing Long-Acting Injectable Cabotegravir to Compete with Generic Oral PrEP,” February 1,2022, *available at* <https://www.massgeneral.org/news/press-release/Study-supports-pricing-long-acting-injectable-cabotegravir-to-compete-with-generic-oral-HIV-PrEP> (last visited March 16, 2022).

[69] PrEP Consumer Group, *supra* note 43.

[70] The team convened two consumer input groups in August and October 2021. The consumer groups included men who were either current or past users of PrEP. Participants included a mix of insurance statuses, race/ethnicity, and geographic location.

[71] Because of recent changes mandating coverage of PrEP medication and ancillary services without cost sharing in most private insurance plans, there is not a need to cover cost sharing on behalf of insured individuals: see Departments of Labor, Health and Human Services, and the Treasury, “FAQs about Affordable Care Act Implementation, Part 47,” July 19, 2021, *available at* <https://www.cms.gov/CCIIO/Resources/Fact-Sheets-and-FAQs/Downloads/FAQs-Part-47.pdf> (last visited March 16, 2022).

[72] 46brooklyn, *supra* note 22.

[73] CDC, *supra* note 20, at 41.

[74] Centers for Disease Control and Prevention, “Vaccines for Children Program (VFC): Eligibility Criteria,” December 17, 2014, *available at* <https://www.cdc.gov/vaccines/programs/vfc/providers/eligibility.html> (last visited March 16, 2022).

[75] Health Resources and Services Administration, “Ryan White HIV/AIDS Program,” December 2020, *available at* <https://hab.hrsa.gov/about-ryan-white-hivaids-program/about-ryan-white-hivaids-program> (last visited February 9, 2022).

[76] K.R. Page , et al., “Promoting Pre-Exposure Prohylaxis to Prevent HIV Infections among Sexual and Gender Minority Hispanics/Latinxs,” AIDS Education and Prevention 29, no. 5 (2017): 389–400; D. Guenter, et al., “Rapid Point-of-Care HIV Testing in Community-Based Anonymous Testing Program: A Valuable Alternative to Conventional Testing,” *AIDS Patient Care and STDs* 22, no. 3 (2008). 18290752 10.1089/apc.2007.0137

[77] Centers for Disease Control and Prevention, “Funding Communities for HIV Prevention,” August 6, 2021, *available at* <https://www.cdc.gov/hiv/policies/strategic-priorities/mobilizing/funding-communities.html> (last visited March 16, 2022); Centers for Disease Control and Prevention, “Eligibility Criteria,” April 11, 2019, *available at* <https://www.cdc.gov/hiv/funding/announcements/ps18-1802/eligibility.html> (last visited March 16, 2022).

[78] This approach could be paired with a mechanism to recoup expenses of high-cost medications for individuals covered by Medicaid from the Medicaid program.

[79] Farnham, *supra* note 2.

[80] This estimate is based on an adjustment to the administrative cost of the Vaccines for Children program (which is approximately $100 million per year in grants to states), an expected $15 million cost for CDC, and an expected $15 million for a vaccine distributor.

[81] CDC, *supra* note 17.

[82] PrEP Consumer Group, *supra* note 43.

[83] V.B. Kirby , et al., The Role of the 340B Drug Pricing Program in HIV-Related Services in California,” Northern California HIV/AIDS Policy Research Center, May 2018, *available at* <https://www.californiaaidsresearch.org/files/340B-HIV-Rapid-Response-Report-FINAL.pdf> (last visited March 16, 2022).

[84] Gilead, “Gilead Announces Updates to The Advancing ACCESS® Patient Assistance/Medication Assistance Program,” April 8, 2021, *available at* <https://www.gilead.com/news-and-press/company-statements/gilead-announces-updates-to-the-advancing-access-patient-assistance-medication-assistance-program> (last visited March 16, 2022).

[85] *Id*.

[86] Kaiser Family Foundation, “The Coverage Gap: Uninsured Poor Adults in States that Do Not Expand Medicaid,” January 21, 2021, *available at* <https://www.kff.org/medicaid/issue-brief/the-coverage-gap-uninsured-poor-adults-in-states-that-do-not-expand-medicaid> (last visited March 16, 2022).

[87] PrEP Consumer Group, *supra* note 43.

[88] Kaiser Family Foundation, “Ending the HIV Epidemic (EHE) Funding Tracker,” February 12, 2021, *available at* <https://www.kff.org/hivaids/issue-brief/ending-the-hiv-epidemic-ehe-funding-tracker> (last visited March 16, 2022).

[89] J. Macrae , “HRSA Releases 2020 Data on HIV Prevention and Treatment in Health Centers,” HIV.gov, September 1, 2021, *available at* <https://www.hiv.gov/blog/hrsa-releases-2020-data-hiv-prevention-and-treatment-health-centers?utm_source=email&utm_medium=email&utm_campaign=daily20210901&utm_content=federalresponse> (last visited March 16, 2022).

[90] J.C. Dombrowski , et al., “Patient Disengagement from an HIV Pre-Exposure Prophylaxis Program in a Sexually Transmitted Disease Clinic,” Sexually Transmitted Diseases 49, no. 9 (2018): e62–e64; Ming, *supra* note 41. 10.1097/OLQ.0000000000000823 PMC6086745 29485544

[91] See for example, AIDSmap, “Six Innovative Models for PrEP Services,” January 7, 2019, available at <https://www.aidsmap.com/news/jan-2019/six-innovative-models-prep-services> (last visited March 16, 2022). See also the HOT4PrEP study which is ongoing, University of Washington, “ID Spotlight: Chase Cannon, MD,” May 28, 2020, available at <https://aid.uw.edu/news/id-spotlight-chase-cannon-md> (last visited March 16, 2022).

